# BICOSS: Bayesian iterative conditional stochastic search for GWAS

**DOI:** 10.1186/s12859-022-05030-0

**Published:** 2022-11-12

**Authors:** Jacob Williams, Marco A. R. Ferreira, Tieming Ji

**Affiliations:** 1 Department of Statistics, Virginia Tech, Blacksburg, 24061 USA; 2 Biostatistics, GRAIL, Menlo Park, 94025 USA

**Keywords:** Bayesian method, GWAS, Model selection

## Abstract

**Background:**

Single marker analysis (SMA) with linear mixed models for genome wide association studies has uncovered the contribution of genetic variants to many observed phenotypes. However, SMA has weak false discovery control. In addition, when a few variants have large effect sizes, SMA has low statistical power to detect small and medium effect sizes, leading to low recall of true causal single nucleotide polymorphisms (SNPs).

**Results:**

We present the Bayesian Iterative Conditional Stochastic Search (BICOSS) method that controls false discovery rate and increases recall of variants with small and medium effect sizes. BICOSS iterates between a screening step and a Bayesian model selection step. A simulation study shows that, when compared to SMA, BICOSS dramatically reduces false discovery rate and allows for smaller effect sizes to be discovered. Finally, two real world applications show the utility and flexibility of BICOSS.

**Conclusions:**

When compared to widely used SMA, BICOSS provides higher recall of true SNPs while dramatically reducing false discovery rate.

**Supplementary Information:**

The online version contains supplementary material available at 10.1186/s12859-022-05030-0.

## Background

Genome wide association studies (GWAS) have been used successfully to identify genes involved with complex traits in a wide variety of species. To identify these genes a statistical analysis is performed to identify which single nucleotide polymorphisms (SNPs) are associated with a trait. The most common form of statistical analysis is single marker analysis (SMA) performed under the mixed model framework [1]. Algorithms such as EMMA [2] (which uses spectral decomposition of the covariance matrix for fast computation), population parameters previously determined (P3D) [3] (which speeds up computation by using the estimates of the variance components from a null model), and EMMAX [4] (which further speeds up computations of EMMA by using the estimate of the heritability from a null model) have led to widespread adoption of the mixed model framework. However, SMA has drawbacks due to not taking into account the correlation structure among SNPs, which leads to high false discovery rate (FDR) and low recall of true causal SNPs [5].

To increase recall and decrease FDR in GWAS, we propose the Bayesian Iterative Conditional Stochastic Search (BICOSS) method. Under a mixed effects model, BICOSS combines Bayesian SMA and Bayesian model selection in an iterative procedure. Each BICOSS iteration has two steps: screening and model selection. BICOSS is initialized with the residuals from a base model that is a linear mixed model with no SNPs. Then the screening step fits as many models as the number of available SNPs, where each model has only one additional SNP and is regressed against the residuals of the base model. This screening step provides a set of candidate SNPs. The second step of BICOSS performs a model search where the possible models contain the base model and any number of SNPs from the set of candidate SNPs. When the model space is too large for complete enumeration, BICOSS performs model selection using Bayesian model selection implemented with a genetic algorithm (GA). The best model found in the model selection step becomes the base model. The next iteration of BICOSS then uses this base model to perform the screening and selection steps. BICOSS iterates between these steps until convergence of the best model. Further details as well as a graphical representation of BICOSS are provided in the “Methods” section. In the "Results" section, a simulation study shows that, when compared to SMA, BICOSS reduces false discovery rate and allows for SNPs with smaller effects sizes to be discovered.

Each iteration of BICOSS conditions on a base model found as the best model in the previous iteration. A key insight gained from our simulation study is that, when compared to SMA, conditioning on SNPs of high importance reduces the error variance thus allowing SNPs with smaller effect sizes to be detected. Other previous works have also used conditional models to find causal SNPs with smaller effect sizes [6–10]. Therefore, by conditioning on SNPs with larger effect sizes found in previous iterations, BICOSS can identify SNPs with smaller effect sizes.

A critical contribution of BICOSS is to combine model selection and screening with conditional models in an iterative procedure. This is important because model selection alone has better FDR control than single marker tests but it tends to have smaller recall. By combining the screening and model selection steps in an iterative procedure, BICOSS consistently increases recall and decreases FDR. To the best of our knowledge, there are only two other GWAS iterative procedures: GWASelect [11] and GWASinlps [12]. Both GWASinlps and GWASelect operate under the simple linear regression framework while BICOSS uses mixed effect regression. GWASelect applies SMA to a large number of bootstrap datasets followed by a LASSO procedure to identify SNPs of interest from conditional models. GWASinlps selects SNPs under a linear regression model using $R^2$. From the set of SNPs, GWASinlps uses Bayesian model selection with nonlocal priors to identify a best SNP model. The two main differences between BICOSS and GWASinlps are that BICOSS uses Bayesian model selection to identify candidate SNPs instead of $R^2$ and BICOSS uses mixed effect models instead of a linear regression models. With the publicly available code for GWASinlps, we compare GWASinlps to BICOSS in the simulation study.

## Methods

BICOSS assumes the general linear mixed model ([1, 2]),

1 $$\begin{aligned} {{\textbf {Y}}} = X {\varvec{\beta }}+ Z {{\textbf {u}}} + {\varvec{\epsilon }}, \quad \text {with} \quad {\varvec{\epsilon }}\sim N(0, \sigma ^2 I) \quad \text {and} \quad {{\textbf {u}}} \sim N(0,\sigma ^2 \tau K), \end{aligned}$$

where ${{\textbf {Y}}}$ is a *n*-dimensional vector of observed phenotypes, *X* is an $n \times p$ matrix with columns including SNPs, intercept, and fixed effects, ${\varvec{\beta }}$ is a *p*-dimensional vector of regression coefficients, *Z* is an $n \times t$ incidence matrix mapping each observed phenotype to one of *t* inbred strains, ${{\textbf {u}}}$ is a *t*-dimensional vector of random effects accounting for population structure, and $\epsilon$ is an error term. In addition, $\sigma ^2$ is the variance of the unstructured error and $\tau$ a kinship dependence parameter. Finally, *K* is the realized relationship matrix or kinship matrix assumed to be a known positive semi-definite matrix.

**Fig. 1:**
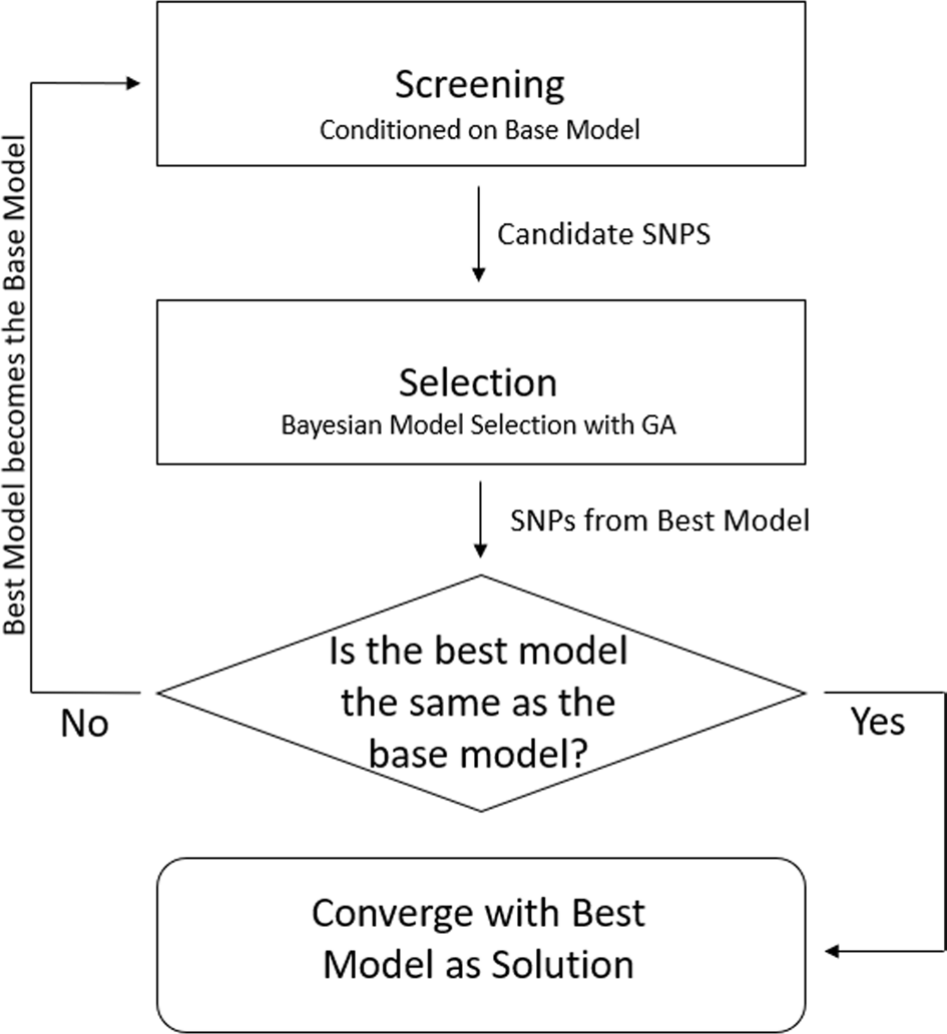
Graphical representation of BICOSS

Figure 1 presents a graphical representation of BICOSS. BICOSS is an iterative procedure where each iteration is comprised of two steps: a screening and a model selection step. BICOSS is initialized with a base model fitted as a linear mixed model with no SNPs in the model. Then the screening step fits as many models as there are SNPs, each model containing one SNP and regressed against the residuals of the base model. The screening step identifies a set of candidate SNPs using Bayesian FDR control applied to the posterior probabilities of the SNPs. Then, the model selection step of BICOSS performs Bayesian model selection where the possible models contain any combination of the base model and SNPs from the candidate set. If the model space is too large to perform complete enumeration, a genetic algorithm is used to perform stochastic model search. The model with the highest posterior probability is the best model. This best model becomes the base model for the next iteration which proceeds with the screening and model selection steps. BICOSS iterates these two steps until convergence of the best model.

We cast both the screening step and model selection step within a Bayesian model selection framework. We briefly highlight Bayesian model selection and the priors on the model space before providing the full derivation for the screening and model selection steps.

### Bayesian model selection

Bayesian model selection assumes *m* possible models $M_1,\dots ,M_m$. Let $P(M_i)$ be the prior model probability for model $M_i$. In addition, assume that the unknown parameters in model $M_i$ are collected in parameter vector $\theta _i \in \Theta _i$ and have prior density $\pi (\theta _i)$. Let the dimension of $\theta _i$ be $d_i$. Finally, assume the likelihood function under model $M_i$ is ${\mathcal {L}}({{\textbf {Y}}}\mid \theta _i, M_i)$. Thus, an important quantity in Bayesian model selection is the marginal likelihood under model $M_i$, $i = 1,\dots , n$, given by

2 $$\begin{aligned} m_i({{\textbf {Y}}}) = \int _{\Theta _i} {\mathcal {L}}({{\textbf {Y}}}\mid \theta _i, M_i) \pi (\theta _i) d\theta _i. \end{aligned}$$

Hence, by Bayes Theorem the posterior probability of model $M_i$ given the data ${{\textbf {Y}}}$ is

3 $$\begin{aligned} P(M_i|{{\textbf {Y}}}) = \frac{P(M_i) m_i({{\textbf {Y}}})}{\sum _{j = 1}^{m} P(M_j) m_j({{\textbf {Y}}})}. \end{aligned}$$

Assuming a base model $M_b$, the Bayes factor of model $M_i$ with respect to $M_b$ is defined as $BF_{ib} = m_i({{\textbf {Y}}})/m_b({{\textbf {Y}}})$. Hence, the posterior probability of model $M_i$ given the data ${{\textbf {Y}}}$ can be computed as

4 $$\begin{aligned} P(M_i|{{\textbf {Y}}}) = \frac{P(M_i) BF_{ib}}{\sum _{j = 1}^{m} P(M_j) BF_{jb}}. \end{aligned}$$

Now, let the BIC of model $M_i$ be

5 $$\begin{aligned} BIC_i = -2 \log \left( {\mathcal {L}}({{\textbf {Y}}}\mid {\hat{\theta }}_i, M_i)\right) + d_i \log (n), \end{aligned}$$

where ${\hat{\theta }}_i$ is the maximum likelihood estimate of $\theta _i$. The Bayes factor $BF_{ib}$ can be approximated by using the BIC ([13, 14]). Specifically, if the information contained in each prior $\pi (\theta _i)$ is equivalent to one observation, then the Bayes factor $BF_{ib}$ can be approximated with

6 $$\begin{aligned} BF_{ib} \approx \text {exp}\left\{ -0.5(BIC_i - BIC_b)\right\} , \end{aligned}$$

with error ${\mathcal {O}}(n^{-1/2})$ [15]. With this approximation, we do not need to explicitly specify the prior densities $\pi (\theta _i)$. BICOSS uses this approximation combined with Eq. 4 to compute the posterior probabilities of the competing models.

### Prior model probabilities in BICOSS

Consider a model with *s* possible SNPs. Following standard practice in modern Bayesian model selection, we treat the inclusion of each of the possible *s* SNPs as independent Bernoulli trials with success probability $(1 - \pi _0)$. As a result, the prior probability of model $M_i$ is

7 $$\begin{aligned} P(M_i) = (\pi _0)^{s - p_i}(1 - \pi _0)^{p_i}, \end{aligned}$$

where $p_i$ is the number of SNPs in model $M_i$. Here, we estimate the true rate of null hypothesis $\pi _0$ using the procedure proposed in [16] which uses the *p* values of each SNP from a SMA to calculate the estimated proportion of true null SNPs. When this procedure conservatively estimates $\pi _0 = 1$, BICOSS sets $\pi _0 = 1 - 100L^{-1}$ where *L* is the total number of SNPs. The *p* values are calculated at every screening step, therefore the estimate of $\pi _0$ is updated at every iteration of the screening step of BICOSS. The model selection step uses the same $\pi _0$ estimated at the first screening, which allows SNPs that were detected in the first screening to be competitive in the model selection compared to SNPs found in subsequent iterations.

### Screening step

The screening step starts by fitting the base model which is obtained from Eq. 1 with the matrix *X* containing the SNPs from the base model of the previous iteration of BICOSS. From this base model fit, we obtain estimates ${\varvec{\hat{\beta }}}$ and ${\hat{\tau }}$. Let $\hat{{{\textbf {Y}}}} = Y - X{\varvec{\hat{\beta }}}$ and let $\Sigma ({\hat{\tau }}) = (I - P)(I + {\hat{\tau }} K)(I - P)$ where $P = X(X^\top (I + {\hat{\tau }}K)^{-1}X)^{-1}X^\top$ is a projection matrix. Recall that *L* is the total number of SNPs. Then the screening step fits for each SNP *l*, $l = 1, \dots , L$, the linear mixed model

8 $$\begin{aligned} \hat{{{\textbf {Y}}}} = X_l \beta _l + {\varvec{\epsilon }}^*, \quad {\varvec{\epsilon }}^* \sim N\left( {{\textbf {0}}},\sigma ^2 \Sigma ({\hat{\tau }})\right) , \end{aligned}$$

where $X_l$ is an $n \times 1$ vector for SNP *l*.

In the screening step, for each SNP *l* we compare only two models: the base model, and the base model with the added SNP *l*. In that context, Eq. 4 in the “Bayesian model selection” section is used to compute the posterior probability of SNP *l* being a causal SNP conditional on the base model. The screening step then scans through all SNPs computing these posterior probabilities.

To control the false discovery rate, BICOSS uses Bayesian FDR control ([17–20]). Let $r_l = 1$ if SNP *l* is a true causal SNP and $r_l = 0$ otherwise. Let $p_l = P(r_l = 1 | {{\textbf {Y}}})$ which is computed as described in the above paragraph using the Bayes factor comparing the model with SNP *l* versus the model without SNP *l*. Then a possible decision rule is to flag SNP *l* as significant if $p_l$ is greater than or equal to a threshold $p_0$. The resulting FDR is then equal to

9 $$\begin{aligned} FDR = \frac{\sum _{l = 1}^L(1 - r_l)1_{p_l \ge p_0}}{\sum _{l = 1}^L1_{p_l \ge p_0}}, \end{aligned}$$

where 1 denotes the indicator function. Further, because the true value of $r_l$ is unknown the posterior expected value of the FDR given the data can be estimated as

10 $$\begin{aligned} {\widehat{FDR}} = \frac{\sum _{l = 1}^L(1 - p_l) 1_{p_l \ge p_0}}{\sum _{l = 1}^L 1_{p_l \ge p_0}}. \end{aligned}$$

A more desired decision rule would be to control for the desired nominal FDR level denoted as $q_0$ rather than an arbitrary predetermined threshold $p_0$. Specifically, we first rank the SNPs in decreasing order of $p_l$. Denote the ordered estimates of the posterior model probabilities as $\{ p_{(1)},p_{(2)},\dots ,p_{(L)} \}$. Thus, denoting $d \in \{ 1,\dots , L \}$, the posterior expected FDR of selecting the first *d* ordered SNPs as significant is

11 $$\begin{aligned} {\widehat{FDR}}_d = \frac{\sum _{l = 1}^L(1 - p_l)1_{p_l> p_{(d)}}}{\sum _{l = 1}^L1_{p_l > p_{(d)}}} = \frac{\sum _{l = 1}^d(1 - p_{(l)})}{d}. \end{aligned}$$

The decision rule for detecting causal SNPs is to flag all SNPs with ${\widehat{FDR}}_d < q_0$. This provides a list of candidate SNPs for the BICOSS selection step. The simulation study and the real data analyses use $q_0 = 0.05$.

### Model selection step

With the list of candidate SNPs from the screening step, the model selection step performs a model search where the possible models include any combination of SNPs in the base model and the candidate SNPs identified in the latest screening. Each possible model is evaluated using the Bayesian model selection procedure described in the “Bayesian model selection” section with prior model probability given in Eq. 7. To accelerate computation, we take a P3D approach and estimate the kinship dependence parameter $\tau$ only once based on the full model that includes the SNPs from the base model as well as the candidate SNPs. This parameter $\tau$ is kept fixed at this estimate when fitting all other models.

Depending on the number of SNPs identified in the screening step, one of two different algorithms are used to search the model space. When the dimensionality is low, a complete enumeration is used to compute posterior model probabilities for every possible model. When the number of SNPs is high such that complete enumeration would be computationally expensive (16 or more), a genetic algorithm is used to search the model space.

BICOSS uses a genetic algorithm implemented in the R package GA [21] that iterates mutation, crossover, and selection steps. The genetic algorithm starts with a population of 100 models. One of these models has just the intercept. Another set of models in this initial population has only one SNP per model, where the SNPs are either from the base model or are candidate SNPs. If there are more than 99 of these SNPs, then the 99 SNPs with the highest posterior probabilities are used to initialize the initial population. If there are less than 99 of these SNPs, then the remaining models in the initial population are chosen at random. The mutation, crossover, and selection steps then operate on the population to create subsequent populations. The mutation step creates a new model from an existing model by changing the status of a SNP in that model, e.g. if a SNP is present in the existing model it will become absent in the new model. The crossover step creates two models by combining two existing models. Finally, the selection step samples models to be passed to the next population with probabilities proportional to $\exp \left( -0.5 BIC_i\right)$ for model $M_i$.

We consider two different convergence criteria, 400 maximum iterations or 40 consecutive iterations with the same best model, whatever happens first. We also considered convergence criteria with 4000 maximum iterations and 400 iterations with the same best model, but the results were about the same. We report the results for the latter set of convergence criteria in the Additional file 1. If the best model identified in the selection step matches the current base model, BICOSS converges. Otherwise, the base model is updated to be the best model found, and another iteration of BICOSS is performed.

## Results

### Simulation study

We have performed a simulation study to compare BICOSS to other competing methods. In addition, we present two smaller simulation studies to evaluate the robustness of BICOSS, when there are no causal SNPs and when there is no kinship dependence structure. For all three simulation studies, we compare BICOSS to SMA methods with the Bonferroni correction and GWASinlps. We consider two SMA methods based on the linear mixed model from Eq. 1: a method we call SMA-Exact that similarly to EMMA uses the spectral decomposition of the kinship dependence structure; and a method we call SMA-Approx. that similarly to EMMAX fixes the variance parameters at their estimates for a model without SNPs. For direct comparison of computation time, all methods are implemented in R. Both BICOSS and SMA methods use a FDR nominal level of 0.05. The genotype data used for all three simulation studies is from 328 *A. Thaliana* accessions from the TAIR9 sequence [22]. In this simulation study $n = 328$ and $Z = I_{n \times n}$. Specifically, we consider a set of 60,000 SNPs. To obtain these 60,000 SNPs, we obtained 10 blocks of 6000 SNPs each with minor allele frequency above 0.01 from *A. Thaliana*, where each block was separated from the subsequent block by 15,000 SNPs. Additional file 1: Fig. S1 presents a heatmap of the correlation matrix of the first block with 6000 SNPs for the 328 *A. Thaliana* accessions. For the general simulation study and the case when there is no kinship dependence structure, we placed the causal SNPs in positions 3000, 9000, 15,000, 21,000, 27,000, 33,000, 39,000, 45,000, 51,000, and 57,000 of the 60,000 SNPs. The kinship matrix used in the case of no causal SNPs and the general simulation study was built from the entire TAIR9 SNP array for the 328 ascensions of *A. Thaliana* using the function A.mat from the R package rrBLUP [23].

We compare the competing methods with four different criteria: recall, also known as true positive rate, FDR, False positive rate, and the F1 score. We also report computation time. Recall is defined as the number of identified true causal SNPs divided by the total number of causal SNPs. The FDR is defined as the number of false positives identified as significant divided by the number of SNPs identified as significant. The false positive rate is the number of false positives divided by the number of false positives plus the number of true negatives. The F1 score is the number of true positives divided by the number of true positives plus half the sum of false positives and false negatives. We report the computation time in seconds for each procedure using 12 cores of a 2 $\times$ 12 core Intel Xeon 2.5 GHz 12-core with 256 GB of memory running OpenBlas for optimized matrix algebra. The results presented here are for GWASinlps version 2.0 with tuning parameters $k_0 = 1$, $n_{skip} = 3$, $r_{xx} = 0.2$, $m = 500$, and $\tau = 0.022$ as recommended in both the GWASinlps documentation and in [12]. For accurate comparison of methods, the results for each simulation setting are based on 100 simulated datasets.

#### General simulation study

A general simulation study to compare BICOSS to other competing methods is conducted under the linear mixed model:

12 $$\begin{aligned} {{\textbf {Y}}} = \alpha {{\textbf {1}}} + X {\varvec{\beta }}+ Z {{\textbf {u}}} + {\varvec{\epsilon }}, \end{aligned}$$

where ${{\textbf {u}}} \sim N(0,\sigma ^2\tau K)$, ${\varvec{\epsilon }}\sim N(0,\sigma ^2 I)$, and $\alpha$ is 1.

We consider 10 causal SNPs with six different settings of $\beta$ vectors. Seven coefficients remained fixed at 0.4 while the other three coefficients were equal to each other and assumed values of 0.05, 0.1, 0.2, 0.4, 0.8, and 1.6. Thus, the fourth setting had equal coefficients across the entire set of causal SNPs. For every simulated ${{\textbf {Y}}}$, the values of $\tau$ and $\sigma ^2$ were equal to 0.1 and 0.2 respectively, which are similar to the estimates of $\tau$ and $\sigma ^2$ obtained in the case study on salt stress in *A. Thaliana*.

**Table 1:** Results of simulation study with linear mixed model

Setting	Measure	Method			
		SMA-exact	SMA-approx.	BICOSS	GWASinlps
	Recall	0.36	0.35	0.49	0.55
Setting 1	FDR	0.61	0.60	0.27	0.62
$\beta ^{(1)} = 0.05$	FPR $\times 10 ^{5}$	12.70	12.30	3.95	17.44
	F1	0.35	0.35	0.57	0.44
	Time (s)	197	2	22	85
	Recall	0.33	0.33	0.49	0.54
Setting 2	FDR	0.57	0.56	0.28	0.61
$\beta ^{(1)} = 0.1$	FPR $\times 10 ^{5}$	11.22	10.90	4.10	16.45
	F1	0.35	0.35	0.57	0.44
	Time (s)	203	2	46	122
	Recall	0.31	0.31	0.49	0.55
Setting 3	FDR	0.61	0.61	0.34	0.63
$\beta ^{(1)} = 0.2$	FPR $\times 10 ^{5}$	11.17	10.87	5.02	19.02
	F1	0.33	0.33	0.55	0.42
	Time (s)	201	2	47	117
	Recall	0.34	0.33	0.58	0.65
Setting 4	FDR	0.59	0.58	0.34	0.62
$\beta ^{(1)} = 0.4$	FPR $\times 10 ^{5}$	10.47	10.22	5.50	20.14
	F1	0.35	0.35	0.61	0.47
	Time (s)	203	2	50	130
	Recall	0.29	0.28	0.73	0.79
Setting 5	FDR	0.79	0.79	0.33	0.60
$\beta ^{(1)} = 0.8$	FPR $\times 10 ^{5}$	21.60	21.35	6.93	22.25
	F1	0.23	0.23	0.69	0.52
	Time (s)	186	1	44	148
	Recall	0.30	0.30	0.70	0.78
Setting 6	FDR	0.92	0.92	0.30	0.65
$\beta ^{(1)} = 1.6$	FPR $\times 10 ^{5}$	61.23	60.49	5.70	25.99
	F1	0.12	0.12	0.69	0.48
	Time (s)	176	1	45	147

Regression coefficients of causal SNPs ${\varvec{\beta }}= (\beta ^{(1)}, 0.4, 0.4, 0.4, \beta ^{(1)}, 0.4, 0.4, 0.4, \beta ^{(1)},0.4)^\top$

Average Performance of each method over 100 datasets for each setting

*Recall* True Positive Rate, *FDR* False Discovery Rate, *FPR* False Positive Rate, *F1* F1 score

Table 1 displays results averaged over the 100 datasets under each setting. SMA procedures typically discover about 3 of the 10 true causal SNPs, BICOSS typically discovers about 5–7 causal SNPs. Therefore, while SMA methods typically discover only the SNPs with large effect sizes, BICOSS is able to discover SNPs with smaller effect sizes. In addition, BICOSS maintains a substantially lower FDR, lower FPR, and higher F1 score in all settings compared to SMA. The massive improvement in these measures is due to the model selection step. Specifically, by allowing multiple SNPs to compete in the best model, BICOSS model selection step better controls FDR.

Compared to GWASinlps, BICOSS provides a similar recall while yielding a much lower FDR, lower FPR, and higher F1 score. BICOSS is more conservative overall than GWASinlps, but the F1 score (that is, the harmonic mean of precision and recall) highlights the improved combined performance in terms of recall and FDR of BICOSS compared to GWASinlps. The better performance of BICOSS when compared to GWASinlps may be explained by two main reasons. First, BICOSS uses a Bayesian screening step while GWASinlps uses a $R^2$-based screening. Second, BICOSS assumes a linear mixed model whereas GWASinlps assumes a linear model with independent errors. In particular, the linear mixed model assumed by BICOSS is more realistic in the context of GWAS analysis.

Our simulation study also shows that when some few SNPs have very large effect sizes as in Settings 5 and 6, SMA methods have difficulty identifying SNPs with medium effect sizes and produce very large FDR. Specifically, Table 1 shows that, in Settings 5 and 6, SMA methods can only find $30\%$ of the true causal SNPs and has FDR of 0.79 and 0.92 respectively. In contrast, in these settings BICOSS has recall at or above $70\%$ and much better FDR control.

#### Robustness to lack of signal

To examine the robustness of BICOSS when applied to datasets with no causal SNPs, we simulate 100 datasets from the model:

13 $$\begin{aligned} {{\textbf {Y}}} = \alpha {{\textbf {1}}}+ Z {{\textbf {u}}} + {\varvec{\epsilon }}, \end{aligned}$$

where ${{\textbf {u}}} \sim N(0,\sigma ^2\tau K)$, ${\varvec{\epsilon }}\sim N(0,\sigma ^2 I)$, and $\alpha = 1$. Similarly, for every simulated ${{\textbf {Y}}}$, the values of $\tau$ and $\sigma ^2$ were equal to 0.1 and 0.2 respectively, which are similar to the estimates of $\tau$ and $\sigma ^2$ obtained in the case study on salt stress in *A. Thaliana*. As there are no true causal SNPs in Eq. 13, we only examine the number of false positives.

**Table 2:** Results of simulation study with no causal SNPs

Setting	Measure	Method			
		SMA-exact	SMA-approx.	BICOSS	GWASinlps
No causal SNPs	FP	0.05	0.04	1.33	8.13
	Time (s)	197	2	22	85

Average Performance of each method over 100 datasets

*FP* the number of false positives

Table 2 presents the results for the 100 simulated datasets under this scenario. In this case, SMA methods have a stricter control of false positives compared to the two iterative procedures. BICOSS performs significantly better than GWASinlps but is not as conservative as SMA. Therefore, one limitation of BICOSS is that it has on average a slightly larger number of false positives then SMA when applied to datasets with no causal SNPs.

#### Robustness to lack of kinship dependence structure

To check how BICOSS performs when the data are from a linear model without kinship dependence, we simulated 100 datasets from the linear model:

14 $$\begin{aligned} {{\textbf {Y}}} = \alpha {{\textbf {1}}} + X {\varvec{\beta }}+ {\varvec{\epsilon }}, \end{aligned}$$

where ${\varvec{\epsilon }}\sim N(0,\sigma ^2 I)$, $\alpha = 1$, and $\sigma ^2 = 0.2$. Note that BICOSS has been built using the mixed model framework. Meanwhile, GWASinlps was built assuming a linear model. Thus, in principle, data simulated from Eq. 14 should favor GWASinlps. We explore one setting of ${\varvec{\beta }}$, all causal coefficients equal to 0.4. Thus, this simulation has identical *X* and ${\varvec{\beta }}$ as setting 4 of the general simulation. *p* values are calculated for SMA using the classic T statistic for simple linear regression models. Therefore as this is an exact procedure we show results labeled as SMA-Exact.

**Table 3:** Results of simulation study with linear model

Setting	Measure	Method		
		SMA-exact	BICOSS	GWASinlps
Linear model	Recall	0.38	0.61	0.66
	FDR	0.62	0.38	0.62
	FPR $\times 10 ^{5}$	12.00	6.95	20.34
	F1	0.37	0.60	0.47
	Time (s)	6	55	169

Regression coefficients of causal SNPs ${\varvec{\beta }}= (0.4, 0.4, 0.4, 0.4, 0.4, 0.4, 0.4, 0.4, 0.4,0.4)^\top$

Average Performance of each method over 100 datasets for each setting

*Recall* True Positive Rate, *FDR* False Discovery Rate, *FPR* False Positive Rate, *F1* F1 score

Table 3 presents the results of the linear model simulation study. Similar to the simulation with linear mixed models, BICOSS has the lowest FDR, lowest FPR, and highest F1. This is not completely surprising because for datasets simulated from Eq. 14, the kinship dependence parameter $\tau$ is usually estimated as very small. In the limit when $\tau$ is estimated to be 0, the linear mixed model in Eq. 1 becomes a linear model. Therefore, even when there is no kinship structure, BICOSS is able to automatically adapt and perform better than competing methods.

### Case studies

To demonstrate the utility and flexibility of BICOSS, we present two case studies with real data analyses. First, BICOSS is implemented on data from a published study of salt stress on the selfing species *A. Thaliana* [24]. Second, BICOSS is applied to a study of alcohol dependency in humans.

**Table 4:** The number of SNPs identified by method for each case study

Method	Salt stress in A. thaliana		AUD in humans	
	Number of SNPs	Time (s)	Number of SNPs	Time (m)
SMA-exact	22	555	15	82
SMA-approx.	22	8	15	4
BICOSS	5	142	6	38
GWASinlps	37	544	499	792

Multiple comparison corrections use nominal level 0.05 and are based on the number of SNPs in a given genotype dataset

#### Salt stress in A. thaliana

This study considers three different settings of soil salt stress to evaluate which genes are potentially impactful [24]. The three settings considered were a control setting, 75 mM of NaCl, and 125 mM of NaCl. Different measures of the root structure were taken to gauge how salt stress impacted the plants. In this case study, we analyze the average length of lateral root per main root length for 328 *A. Thaliana* accessions under 75 mM NaCl salt stress. Genotype data was obtained from TAIR9 [22]. Only SNPs with minor allele frequency greater than 0.01 were included, thus the analysis presented here considers approximately 213,000 SNPs.

Table 4 presents the number of SNPs found by SMA, BICOSS, and GWASinlps as well as the computational time. For *A. Thaliana*, both SMA methods found 22 SNPs, GWASinlps found 37 SNPs, and BICOSS identified just 5 SNPs. Similar to the simulation study, we see a large difference in the total number of SNPs found by BICOSS when compared to SMA and GWASinlps. Surprisingly, we note a large increase of the total number of SNPs found by GWASinlps compared to SMA. Given the results of the simulation study, we expect the majority of SNPs found by GWASinlps and SMA methods to be false positives. Based on the simulation study, BICOSS has a much better control of FDR than the other methods. Thus, for purpose of discussion we will focus on the results from BICOSS. Of the five SNPs identified by BICOSS, one SNP is perfectly correlated to two other SNPs, implying seven identified SNPs.

The seven SNPs are in genes AT1G62500, AT2G38970, AT3G60370, AT4G14305, AT4G39955, AT4G39970, and AT4G40000. Previous literature relates two of these genes to response to salt stress. Specifically, AT1G62500 is a differentially expressed gene which has been shown to activate in the event of salt stress [25]. In addition; AT4G39955 is an $\alpha$/$\beta$-Hydrolases superfamily protein. $\alpha$/$\beta$-Hydrolases superfamily proteins have been shown to enhance salt tolerance in the sweet potato family [26].

#### Alcohol use disorder in humans

In this case study, we use publicly available data from The Collaborative Study on the Genetics of Alcoholism (COGA) that was performed to identify novel genetic factors associated with alcohol use disorder (AUD) [27]. Specifically, in this case study we analyze the response variable “age of first drink”, for 1738 people of European ancestry with approximately 1 million sequenced SNPs. To normalize and variance-stabilize the data, the logarithm transformation was applied to age of first drink. Only SNPs with minor allele frequency larger than 0.01 were investigated for this analysis. Further, any SNP that did not have an rsID or was located in chromosome X or Y was removed from the analysis. Thus, this analysis considers approximately 840,000 SNPs.

Table 4 presents the number of SNPs found by SMA, BICOSS and GWASinlps and the timing of each method. Similarly to the simulation study and the *A. Thaliana* case study, SMA and GWASinlps identified large numbers of SNPs. Specifically for the AUD case study, both SMA methods found 15 SNPs, GWASinlps found 499 SNPs, and BICOSS found just 6 SNPs. Because BICOSS has a much better FDR control than the other methods, here we investigate the genes found by BICOSS. BICOSS identified six SNPs, which are in the following genes: KCNMA1, ZYG11A, TPTE2, ABCF1, ANKS1B, and LINC02237. LINC02237 is a long intergenic non-protein coding RNA and the other genes are all protein coding genes.

Of the five protein coding genes found by BICOSS, two have published associations with AUD and two have been linked to liver diseases. Specifically, KCNMA1 is known as a gene associated with alcohol dependency [28]. In addition, in a study with people of Chinese Han ethnicity, ANKS1B has been found to be associated with alcoholism [29]. Further, TPTE2 has been shown to be related to hepatic fibrogenesis and fibrosis [30]; alchohol abuse is one of the main causes of liver fibrosis [31]. Furthermore, ABCF1 has been shown to be overexpressed in hepatocellular carcinoma [32]. These results indicate possibly important genes for further potential investigation for a better understanding of alcohol use disorder.

## Discussion

We have presented BICOSS, a novel Bayesian method for the analysis of GWAS data. To take into account the correlation structure among SNPs, BICOSS iterates a screening step and a model selection step. Simulation studies show that, while when there are no true SNPs BICOSS tends to identify a slightly larger number of SNPs than SMA methods, when there are true causal SNPs, BICOSS performs much better than SMA. In the latter case when compared to SMA, BICOSS has greater recall of true causal SNPs while maintaining a much lower FDR. In addition, when there are SNPs with large effect sizes, BICOSS has increased recall of true causal SNPs with small and medium effect sizes. Further, when compared to the Bayesian iterative method GWASinlps, BICOSS maintains comparable recall while having a much lower FDR.

While here we have implemented BICOSS within the EMMAX [4] methodology, we note that BICOSS can be easily adapted to work with other GWAS frameworks such as GCTA [33]. Applying BICOSS should be relatively straightforward when the model and the likelihood can be explicitly written.

There are many possible avenues for future research. For example, a potentially useful avenue is to extend BICOSS to use explicit prior distributions for the parameters. Such extension would allow the incorporation of substantive prior information in the GWAS analysis. Another possible area of research would be to extend BICOSS to BioBank scale data. Finally, another possible area of research would be to extend BICOSS for the analysis of non-Gaussian data such as the number of lateral roots in *A. Thaliana* or the indicator of alcohol dependency for families with members suffering alcohol use disorder.

## Conclusion

We propose BICOSS, a novel iterative Bayesian procedure for GWAS analysis. Compared to SMA, BICOSS increases recall of true causal SNPs while dramatically reducing FDR. Upon publication of this article, BICOSS will be made available in the R package GWAS.BAYES that is available of Bioconductor.

## Supplementary Information

**Additional file 1**. Additional simulation study results and correlation matrix of the first 6,000 SNPs used in the simulation study.

## Data Availability

The two case study datasets are publicly available from the following websites. A. Thaliana phenotype: https://arapheno.1001genomes.org; A. Thaliana genotype: dataset available from R package qtcat.data (https://rdrr.io/github/QTCAT/qtcat.data/; and genotype and phenotype data for Alcohol use disorder in humans: www.ncbi.nlm.nih.gov/projects/gap/cgi-bin/collection.cgi?study_id=phs000092.v1.p1.

## Abbreviations

SMA: Single marker analysis
GWAS: Genome-wide association studies
SNPs: Single nucleotide polymorphisms
BICOSS: Bayesian iterative conditional stochastic search
FDR: False discovery rate
GA: Genetic algorithm
BIC: Bayesian information criterion
MAF: Minor allele frequency
