# Supplementary material for: BICOSS: Bayesian iterative conditional stochastic search for GWAS
Source: BMC Bioinformatics. 2022 Nov 12;23:475. doi: 10.1186/s12859-022-05030-0 (PMC9652902; doi:10.1186/s12859-022-05030-0)
Supplement: Supplementary file 1 — Additional file 1. Additional simulation study results and correlation matrix of the first 6,000 SNPs used in the simulation study. [file 12859_2022_5030_MOESM1_ESM.pdf]

Supplementary Material for BICOSS: Bayesian Iterative Conditional Stochastic Search for GWAS

1 Comparison of BICOSS with different Convergence Criterion

BICOSS has two different criteria for convergence of the genetic algorithm, maximum number of iterations and number of consecutive iterations at the same best model. Below there are three tables for each simulation study of the manuscript comparing BICOSS with 400 maximum iterations and 40 consecutive iterations to BICOSS with 4000 maximum iterations and 400 consecutive iterations.

1.1 General Simulation

The general simulation study looked at 6 different sets of coefficients labeled as settings. The full details of the simulation study are provided in the manuscript.

1.2 Robustness to Lack of Signal

The Robustness to Lack of Signal simulation study investigate how BICOSS performs when there are no causal SNPs.

1.3 Robustness to Lack of Kinship Dependence Structure

The robustness to lack of kinship dependence structure investigates how BICOSS performs when the data is simulated from a linear model instead of a linear mixed model.

2 Correlation of First Set of SNPs

Figure S1 is a correlation heatmap of the first 6,000 SNPs used in the simulation study of the main text as well as the supplementary material.

Table S1: Results of general simulation with linear mixed model comparing BICOSS convergence criterion 400 maximum iterations and 40 consecutive iterations at the same best model (400/40) and 4000 maximum iterations and 400 consecutive iterations at the same best model (4000/400). Regression coefficients of causal SNPs $\boldsymbol{\beta} = (\beta^{(1)}, 0.4, 0.4, 0.4, \beta^{(1)}, 0.4, 0.4, 0.4, \beta^{(1)}, 0.4)^\top$. Average Performance of each method over 100 datasets for each setting. Recall indicates the True Positive Rate, FDR is the False Discovery Rate, FPR is the False Positive Rate, and F1 is the F1 score.

Setting	Measure	Method	
		BICOSS 400/40	BICOSS 4000/400
Setting 1 $\beta^{(1)} = 0.05$	Recall	0.49	0.50
	FDR	0.27	0.27
	FPR $\times 10^5$	3.95	3.97
	F1	0.57	0.58
	Time (s)	46	51
Setting 2 $\beta^{(1)} = 0.1$	Recall	0.49	0.49
	FDR	0.28	0.29
	FPR $\times 10^5$	4.10	4.15
	F1	0.57	0.57
	Time (s)	47	53
Setting 3 $\beta^{(1)} = 0.2$	Recall	0.49	0.49
	FDR	0.34	0.33
	FPR $\times 10^{-5}$	5.02	4.87
	F1	0.55	0.55
	Time (s)	50	56
Setting 4 $\beta^{(1)} = 0.4$	Recall	0.58	0.59
	FDR	0.34	0.33
	FPR $\times 10^5$	5.50	5.57
	F1	0.61	0.61
	Time (s)	44	62
Setting 5 $\beta^{(1)} = 0.8$	Recall	0.73	0.74
	FDR	0.33	0.32
	FPR $\times 10^5$	6.93	6.63
	F1	0.69	0.70
	Time (s)	45	70
Setting 6 $\beta^{(1)} = 1.6$	Recall	0.70	0.71
	FDR	0.30	0.30
	FPR $\times 10^5$	5.70	5.58
	F1	0.69	0.69
	Time (s)	68	59

Table S2: Results of simulation with no causal SNPs comparing BICOSS with convergence criterion 400 maximum iterations and 40 consecutive iterations at the same best model (400/40) and 4000 maximum iterations and 400 consecutive iterations at the same best model (4000/400). Regression coefficients of causal SNPs $\boldsymbol{\beta} = (0.4, 0.4, 0.4, 0.4, 0.4, 0.4, 0.4, 0.4, 0.4, 0.4)^\top$ Average Performance of each method over 100 datasets. FP indicates the the number of False Positives.

Setting	Measure	Method	
		BICOSS 400/40	BICOSS 4000/400
No Causal SNPs	FP	1.33	1.32
	Time (s)	22	22

Table S3: Results of simulation with linear model comparing BICOSS with convergence criterion 400 maximum iterations and 40 consecutive iterations at the same best model (400/40) and 4000 maximum iterations and 400 consecutive iterations at the same best model (4000/400). Average Performance of each method over 100 datasets for each setting. Recall indicates the True Positive Rate, FDR is the False Discovery Rate, FPR is the False Positive Rate, and F1 is the F1 score.

Setting	Measure	Method	
		BICOSS 400/40	BICOSS 4000/400
Linear Model	Recall	0.61	0.60
	FDR	0.38	0.39
	FPR $\times 10^5$	6.95	7.30
	F1	0.60	0.60
	Time (s)	55	48

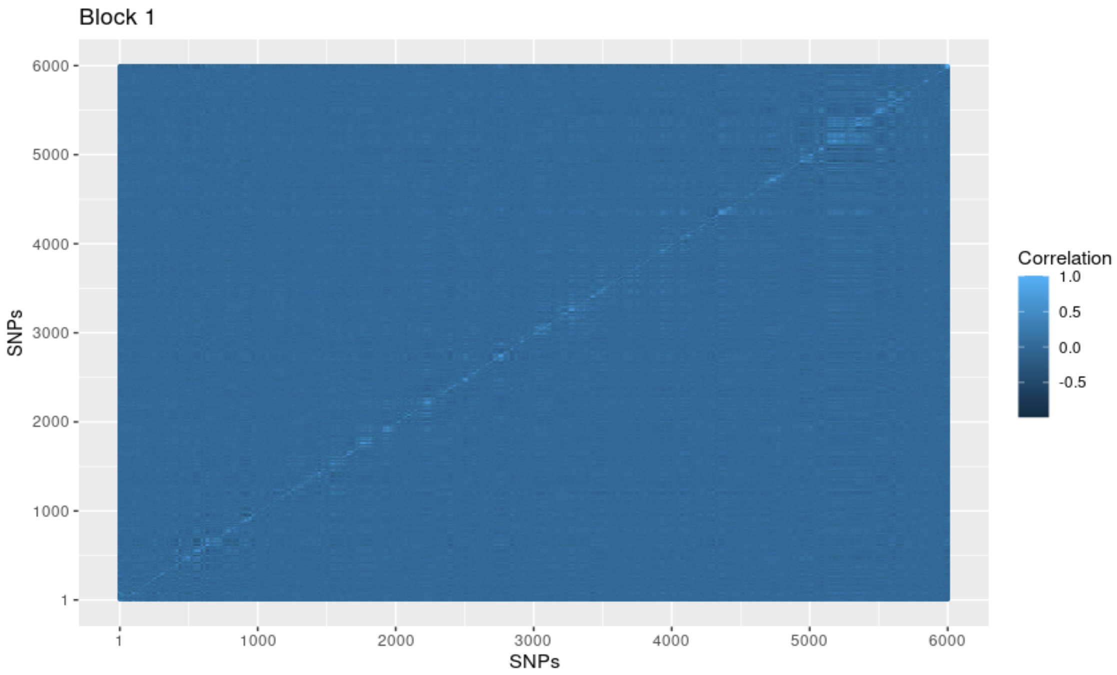

Figure S1: Correlation Plot of Block 1 for the Simulation Studies.
